# Rationale and design of the CONFIRM2 (Quantitative COroNary CT Angiography Evaluation For Evaluation of Clinical Outcomes: An InteRnational, Multicenter Registry) study

**DOI:** 10.1016/j.jcct.2023.10.004

**Published:** 2023-11-10

**Authors:** Alexander R. van Rosendael, Tami Crabtree, Jeroen J. Bax, Rine Nakanishi, Saima Mushtaq, Gianluca Pontone, Daniele Andreini, Ronny R. Buechel, Christoph Gräni, Gudrun Feuchtner, Toral R. Patel, Andrew D. Choi, Mouaz Al-Mallah, Faisal Nabi, Ronald P. Karlsberg, Carlos E. Rochitte, Mirvat Alasnag, Ashraf Hamdan, Filippo Cademartiri, Hugo Marques, Dinesh Kalra, David M. German, Himanshu Gupta, Martin Hadamitzky, Roderick C. Deaño, Omar Khalique, Paul Knaapen, Udo Hoffmann, James Earls, James K. Min, Ibrahim Danad, The CONFIRM 2 investigators

**Affiliations:** a Department of Cardiology, Leiden University Medical Center, Leiden, The Netherlands; b Cleerly, Inc, Denver, CO, United States of America; c Department of Cardiovascular Medicine, Toho University Graduate School of Medicine, Tokyo, Japan; d Department of Perioperative Cardiology and Cardiovascular Imaging, Centro Cardiologico Monzino IRCCS, Milan, Italy; e Department of Biomedical, Surgical and Dental Sciences, University of Milan, Milan, Italy; f Division of University Cardiology, IRCCS Galeazzi Sant’ Ambrogio, Department of Biomedical and Clinical Sciences, University of Milan, Italy; g Department of Nuclear Medicine, Cardiac Imaging, University Hospital and University of Zurich, Zurich, Switzerland; h Department of Cardiology, Inselspital, Bern University Hospital, University of Bern, Bern, Switzerland; i Department of Radiology, Medical University of Innsbruck, Innsbruck, Austria; j Cardiology at Stroobants Heart and Vascular Institute and UVA Cardiology, Lynchburg, VA, United States of America; k Cardiology and Radiology, George Washington University, Washington, DC, United States of America; l Department of Cardiology, Houston Methodist, Houston, TX, United States of America; m Cardiovascular Research Foundation of Southern California, Cedars Sinai Heart Institute, David Geffen School of Medicine, University of California Los Angeles, Los Angeles, CA, United States of America; n Heart Institute, InCor, University of São Paulo Medical School, São Paulo, Brazil; o Cardiac Center, King Fahd Armed Forces Hospital, Jeddah, Saudi Arabia; p Department of Cardiology, Rabin Medical Center, Petah Tikva, Israel; q Department of Imaging, Fondazione Monasterio/CNR, Pisa, Italy & SYNLAB IRCCS SDN, Naples, Italy; r UNICA, Unit of Cardiovascular Imaging, Hospital da Luz, Lisboa and Católica Medical School, Portugal; s Division of Cardiology, Department of Medicine, University of Louisville School of Medicine, Louisville, KY, United States of America; t Knight Cardiovascular Institute, Oregon Health & Science University, Portland, OR, United States of America; u Cardiac Imaging, Heart and Vascular Institute, Valley Health System, Ridgewood, NJ, United States of America; v Department of Radiology and Nuclear Medicine, German Heart Center Munich, Munich, Germany; w Department of Medicine, Division of Cardiovascular Medicine, University of Wisconsin School of Medicine and Public Health, Madison, WI, United States of America; x Division of Cardiovascular Imaging, St. Francis Hospital & Heart Center, Roslyn, NY, United States of America; y Department of Cardiology, Amsterdam University Medical Center, Location VUMC, Amsterdam, The Netherlands; z Department of Cardiology, University Medical Center Utrecht, Utrecht, The Netherlands

**Keywords:** Computed tomography angiography, Atherosclerosis, Prognosis, Coronary artery disease, Artificial intelligence, Machine-learning

## Abstract

**Background:**

In the last 15 years, large registries and several randomized clinical trials have demonstrated the diagnostic and prognostic value of coronary computed tomography angiography (CCTA). Advances in CT scanner technology and developments of analytic tools now enable accurate quantification of coronary artery disease (CAD), including total coronary plaque volume and low attenuation plaque volume. The primary aim of CONFIRM2, (Quantitative **CO**ro**N**ary CT Angiography Evaluation **F**or Evaluation of Clinical Outcomes: An **I**nte**R**national, **M**ulticenter Registry) is to perform comprehensive quantification of CCTA findings, including coronary, non-coronary cardiac, non-cardiac vascular, non-cardiac findings, and relate them to clinical variables and cardiovascular clinical outcomes.

**Design:**

CONFIRM2 is a multicenter, international observational cohort study designed to evaluate multidimensional associations between quantitative phenotype of cardiovascular disease and future adverse clinical outcomes in subjects undergoing clinically indicated CCTA. The targeted population is heterogenous and includes patients undergoing CCTA for atherosclerotic evaluation, valvular heart disease, congenital heart disease or pre-procedural evaluation. Automated software will be utilized for quantification of coronary plaque, stenosis, vascular morphology and cardiac structures for rapid and reproducible tissue characterization. Up to 30,000 patients will be included from up to 50 international multi-continental clinical CCTA sites and followed for 3–4 years.

**Summary:**

CONFIRM2 is one of the largest CCTA studies to establish the clinical value of a multiparametric approach to quantify the phenotype of cardiovascular disease by CCTA using automated imaging solutions.

## 1. Introduction

Atherosclerosis is the primary cause of coronary heart disease and can remain asymptomatic for many years with an acute coronary syndrome (ACS) or sudden cardiac death often being its first clinical manifestation.^1^ Therefore, coronary artery disease (CAD) is a daunting problem which has spurred a relentless search for the ‘Holy Grail’, namely precise prediction of ACS using non-invasive imaging techniques such as coronary computed tomography angiography (CCTA). The unique characteristics of CCTA enable direct visualization of atherosclerotic plaques and numerous studies showed its strength in excluding “significant” CAD with a near to absolute certainty. Therefore, contemporary global guidelines advocate the use of CCTA in symptomatic patients using ≥ 50% angiographic stenosis (i.e. obstructive CAD) to reflect an increased likelihood of ischemia-causing lesions.^2^ Interestingly, a concise and elegant parameter such as an angiographic stenosis ≥ 50% by CCTA appears also a strong predictor of future major adverse cardiac events (MACE) and mortality.^3^

Much of the multicenter clinical data that supports the prognostic importance of CCTA findings of CAD was generated by CONFIRM, or the **CO**ro**N**ary CT Angiography Evaluation **F**or Evaluation of Clinical Outcomes: An **I**nte**R**national, **M**ulticenter Registry.^4^ The CONFIRM study investigated the incremental prognostic value of CCTA beyond traditional risk factors in 23,854 individuals undergoing CCTA for prediction of MACE and all-cause mortality demonstrating ≥50% stenosis as a strong predictor of future mortality at 2.3 years, and a dose-response relationship to increased risk based upon the number of severe stenoses in coronary arterial distributions.^5^ Furthermore, sex differences were observed, with women developing CAD approximately 10 years later and exhibiting higher risk of mortality than their male counterparts for 2-vessel and 3-vessel/left main disease.^5,6^ Since the initial publications of this study, numerous additional studies have evaluated the prognostic strength of CCTA findings in different subgroups, noting consistently improved risk stratification with CCTA in patients without known CAD, as well as with known CAD, with coronary artery calcium scores = 0, with no treatable CAD risk factors, with diabetes, at age <45 years, with family history of premature CAD, with left ventricular dysfunction, with chronic total occlusions, with chronic kidney disease and post-coronary artery bypass grafting (CABG) and percutaneous coronary intervention (PCI).^5,7–14^

Beyond assessment of stenosis severity, CCTA also enables assessment of coronary atherosclerotic phenotype. Certain plaque features such as positive remodeling, low attenuation plaque, spotty calcifications and the napkin ring sign are the basis of ‘high risk’ plaques which are independent predictors of cardiovascular events and improve risk prediction beyond mere stenosis assessment.^15–19^ Indeed, the landmark SCOT-HEART (Scottish Computed Tomography of the HEART) trial found low attenuation plaque volume quantified from CCTA to provide the most powerful predictive information for future myocardial infarction independent from clinical risk factors, coronary calcium score and stenosis severity.^19^ Despite these great achievements, the prognostic value of a quantitative phenotype of CAD has not been fully exploited. Furthermore, semi-quantitative plaque analyses are time-consuming, require a high level of experience, and are not widely performed. Artificial intelligence (AI) and machine-learning (ML) approaches are increasingly utilized to overcome this barrier, allowing the extraction of quantitative plaque measures such as total plaque volume as well as the volume of plaque subtypes (calcified, partially calcified, non-calcified, fibrous, fibrofatty and low attenuation plaque), length of lesions and numerous other features which may not be visible to the naked eye.^20–22^ CCTA further allows quantification of the whole heart including cardiac chamber sizes, left ventricular mass, or left ventricular scar, all of which have shown clinical and prognostic significance. We will perform combined coronary artery evaluation and cardiac structural evaluation by CCTA, and evaluate the incremental prognostic value.

Therefore, the aim of the CONFIRM2 study is to quantify coronary plaque, stenosis, vascular morphology and cardiac structures to allow for a comprehensive analysis of CCTA images and evaluate multidimensional associations between phenotypic manifestations of cardiovascular disease, demographic and clinical information, and outcomes. We aim to improve risk prediction and provide a platform for precision medicine in patients undergoing CCTA.

## 2. Methods

### 2.1. Overall study design

CONFIRM2 is a multicenter, international, observational cohort study. The clinical implications of CCTA findings including coronary artery disease (e.g., atherosclerosis, stenosis, ischemia, etc), non-coronary cardiac findings (e.g., myocardial, valvular, pericardial, etc), non-cardiac vascular findings (e.g., aortic, pulmonary artery, pulmonary veins, etc), and non-cardiac findings (e.g., emphysema, bone mineral density, etc) will be evaluated. The time-related nature of CCTA will be explored, including cross-sectional (e.g., clinical presentation, baseline laboratory values), time-varying (e.g., changes in clinical presentation, medications and laboratory values over time), longitudinal (e.g., risk prediction for clinical events, treatment effect), and interdependence (e.g., relationship of LA volumes to CAD to epicardial fat) relationships of different CT parameters. Different analytic approaches will include quantitative (e.g., quantitative FDA-cleared software for CCTA analysis), machine learning (e.g., deep learning-based approaches), and radiomics for their value to detect “significant” disease (e.g. hemodynamic significant CAD) and as prognosticator.

### 2.2. Study objectives

#### 2.2.1. Primary study objective

The primary study objective of the CONFIRM2 is to evaluate the prognostic value of quantitative measures of coronary atherosclerotic burden, stenosis, vascular and plaque morphology for prediction of death, myocardial infarction and cerebrovascular events. Machine learning techniques and Cox proportional hazard models will be used to evaluate the selection of the most prognostic value of variables.

#### 2.2.2. Secondary objectives

Secondary objectives include further exploration of the clinical value, prognostic value, and categorization into risk groups of individual atherosclerotic plaque features. Secondary studies will aim to optimize the definition of ‘high-risk plaque features’, determine the associations between whole-heart atherosclerosis evaluation and clinical symptoms, determine the incremental value beyond clinical variables or coronary artery calcium score, evaluate of novel methodologies of the coronary stenosis severity assessment, evaluate prognostic value of vascular morphology (lumen volume, or vessel volume), etc. The time related nature of clinical risk factors, laboratory values or medication usage over time will be evaluated in association with coronary atherosclerotic phenotype. Studies will evaluate associations of plaque with novel antiatherosclerotic medications or serum laboratory markers, and evaluate non common clinical risk factors (menopause, sedentary lifestyle, diet, etc). Associations with myocardial ischemia by several invasive or non-invasive techniques will be evaluated. The clinical value of non-coronary structures such as (peri-coronary) adipose tissue, heart chamber quantification, LV wall characterization, valve assessment will be evaluated for several clinical outcomes, including valve replacement or repair, or other cardiac interventions.

### 2.3. Study endpoints

The primary endpoint of CONFIRM2 is the time to first occurrence of all-cause death.

Secondary endpoints include major adverse cardiac events (MACE), defined by the occurrence of any of the following events:

- All-cause death
- *Myocardial infarction*, defined in accordance to the Universal Definition of Myocardial Infarction as established by the European Society of Cardiology (ESC), American College of Cardiology (ACC), American Heart Association (AHA), and the World Heart Federation.
- *Cerebrovascular accident*, defined as a neurological deficit lasting ≥24 h or lasting <24 h with a brain imaging study showing infarction.

Tertiary endpoints include:

- *Late coronary revascularization*, defined as revascularization ≥90 days following the index diagnostic test.
- *Valvular intervention*, defined as surgical or transcatheter valve replacement or repair of any cardiac valve.
- *Congestive heart failure (CHF),* defined by the Framingham Heart Study criteria, and will be made when 2 major criteria are present or 1 major and 2 minor criteria are present concurrently.
- *Cardiac death*, defined as due to any of the following: 1) myocardial infarction; 2) heart failure (includes death due to CHF, cardiogenic shock or pulmonary edema. All deaths from hypotension (systolic blood pressure <80 mmHg) and/or respiratory failure without other clear etiology should be classified as heart failure; 3) arrhythmic death; 4) unclear cause of death, but cardiac cause cannot be excluded.

### 2.4. Targeted population

This study will enroll up to 30,000 patients undergoing clinically indicated CCTA and follow-up for 3–4 years. This number is based on empirical data to provide adequate data to permit subgroup analyses such as for sex, ethnicity and cardiovascular risk factor categories in relation to coronary atherosclerosis. Sites participating in the study will be selected based on data quality and quantity of CCTAs per year, and to achieve adequate representation of race and ethnicity. The study is considered non-significant risk because this is an observational registry with no targeted downstream alteration to the clinical care pathway of the patient or additional interventions. CCTA, in both the US (ACC/AHA) and European guidelines (ESC, NICE guidelines), received a class I indication for the use as a first-line test in chest pain patients.^2,23^

### 2.5. Eligibility criteria

#### 2.5.1. Patient eligibility criteria

Patients referred for clinically indicated CCTA will include those undergoing evaluation for CAD, valvular heart disease or congenital heart disease, and pre-procedural evaluation for CABG, PCI, surgical aortic valve replacement/repair (SAVR), transcatheter aortic valve implantation (TAVI), left atrial appendage (LAA) occlusion, surgical mitral valve replacement/repair (SMVR), transcatheter mitral valve replacement (TMVR), tricuspid valve interventions, patent foramen ovale (PFO) closure, electrophysiological (EP) intervention including pre-pulmonary vein isolation (PVI). All consecutive patients who have presented or are presenting for clinically-indicated CCTA of ≥64-detector rows and who meet all the inclusion criteria and none of the exclusion criteria will be included into CONFIRM2. Further inclusion and exclusion criteria are described in Table 1.

#### 2.5.2. Site eligibility criteria

Each clinical center is required to obtain IRB approval for the protocol and consent (and their revisions) in a timely fashion, to recruit patients, to collect data and enter it accurately in the electronic data capture (EDC) system, to faithfully follow the protocol and adhere to the standards of Good Clinical Practice (GCP). Each participating site contributing patient data to CONFIRM2 fulfills the following site requirements: (1) greater than 500 patients per annum undergoing CCTA by ≥ 64-detector rows, (2) able to perform follow up on recruited patients, (3) able to organize data required for completion of CONFIRM2 case report form, and (4) able to perform de-identification of Protection Health Information (PHI) securely on-site in a manner in keeping with local and international regulations. In addition to site specific IRB evaluation, central IRB approval of CONRIFM2 was obtained.

### 2.6. Study sites/participating centers

Since the recruitment of patients started in 2021, 20 sites have contributed data to the CONFIRM2 from 11 countries (United States, Japan, The Netherlands, Italy, Switzerland, Austria, Saudi Arabia, Israel, Portugal, Brazil, and Germany). The dynamic nature of the study allows further inclusion of sites in the upcoming years. Qualifying patients will be enrolled, or retrospectively included in the study and followed for 3–4 years through follow up visits and/or phone calls. Up to 50 medical centers from US and out of US will participate in the study, with up to 30,000 subjects entered. Each center may not enroll more than 1000 – 3000 subjects. The geographic clusters were selected to represent medical centers of different sizes with different diagnostic capabilities, as well as differences in clinical and demographical patient populations. The medical centers all have incorporated CCTA into their daily clinical practice.

### 2.7. Patient evaluation and follow-up

#### 2.7.1. Patient evaluation

At baseline, clinical, demographical, laboratory, and imaging data will be collected and entered into the case report form. The indication for CCTA includes: CAD evaluation (symptomatic stable; symptomatic acute; asymptomatic [abnormal stress or other], asymptomatic screening), asymptomatic pre-operative evaluation, TAVI, LAA occlusion, TMVR, tricuspid valve interventions, pulmonary vein ablations, electrophysiological evaluation, congenital heart disease, or other. The following laboratory assessment will be collected: serum creatinine, total cholesterol, LDL-C, HDL-C, triglycerides, HbA1C, CRP, Lp(a), Troponin, BNP. Patient demographics include age, sex, ethnicity/race, height, weight, and vital signs. Patient history of CAD includes prior myocardial Infarction (MI), PCI, and CABG. Concomitant medications will be collected: antiplatelet agents, oral anticoagulants, anti-hypertensives, anti-arrhythmics, Hairt failure medications, lipid lowering agents, diabetic agents, smoking cessation agents, anti-depressants, and pulmonary medications. Risk factors include hypertension, dyslipidemia, diabetes, family history, smoking, sedentary lifestyle, peripheral arterial disease, gestational hypertension, gestational diabetes and menopause. Hypertension was defined as a blood pressure of ≥140/90 mmHg or the use of antihypertensive medication. Dyslipidemia was defined as a total cholesterol level of ≥5 mmol/L or treatment with cholesterol-lowering medication. Patients were classified as having diabetes if they were receiving treatment with oral hypoglycemic drugs or insulin. A positive family history of CAD was defined by the presence of CAD in first-degree relatives younger than 55 years in men or 65 years in women. Imaging data (including actual DICOM images) besides the CCTA will be also collected such as a nuclear myocardial perfusion imaging, CMR stress perfusion imaging, invasive coronary angiography, fractional flow reserve (FFR), optical coherence tomography, and intra vascular ultra sound (IVUS).

#### 2.7.2. Follow-up

**Fig. 1.**
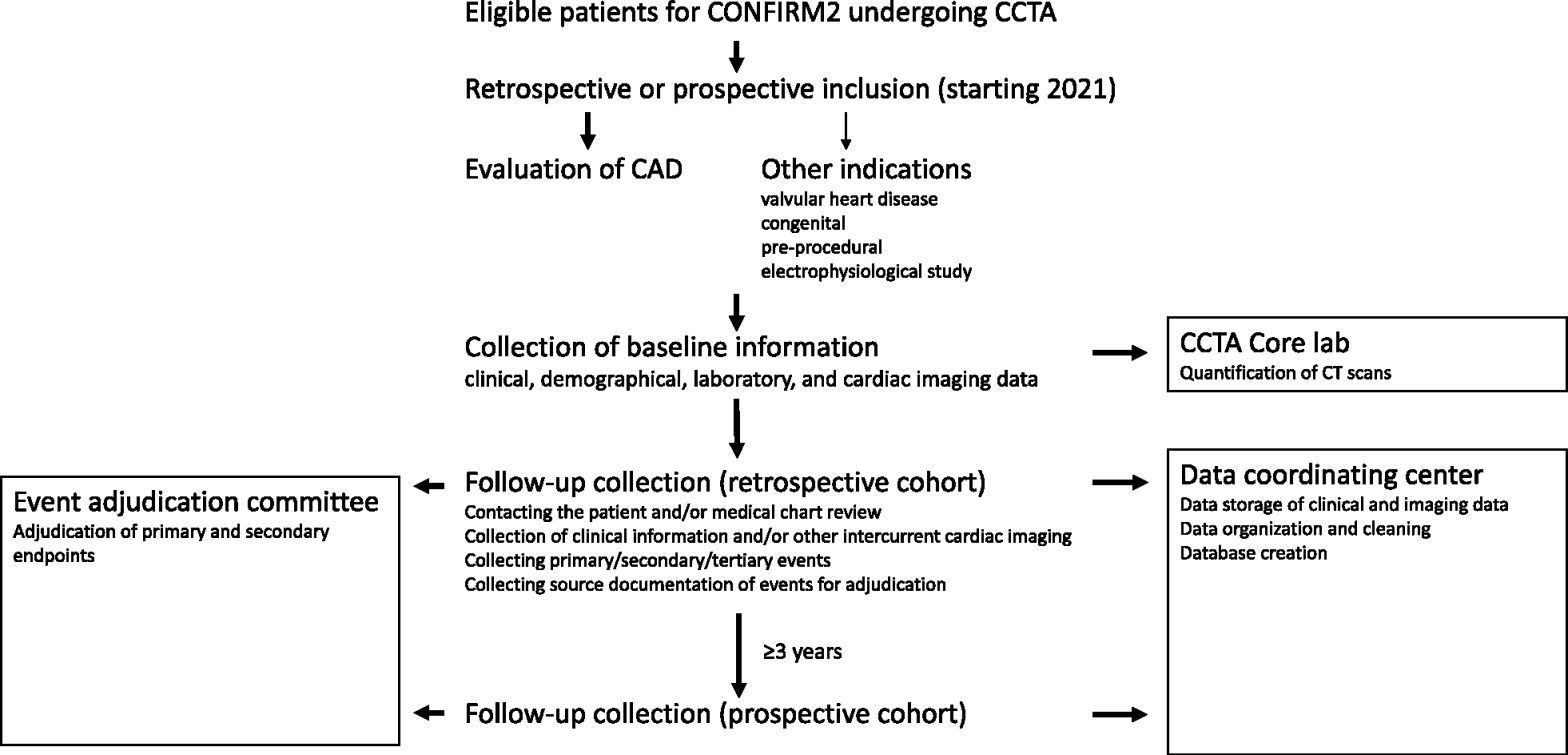
Flowchat.

During periodical follow-up assessment, the post-CCTA medical treatment, other performed imaging tests (nuclear myocardial perfusion imaging, CCTA, invasive coronary angiography, FFR, or IVUS), clinical presentation (symptoms, pre-test likelihood of CAD), laboratory results, medication usage and dose will be collected to enable longitudinal assessment of risk during the study period. The occurrence of study endpoints (as mentioned previously) will be assessed by the sites during follow-up. The participating sites will submit clinical information including baseline and event electrocardiograms, troponin trends, the discharge summary, invasive angiogram images and clinical reports, brain imaging, and neurology consult reports to the core laboratory for adjudication of the primary and secondary endpoint. An independent event adjudication committee will adjudicate events. A flow chart from patient inclusion to follow-up collection is in Fig. 1.

### 2.8. Acquisition and interpretation of CCTA

**Fig. 2.**
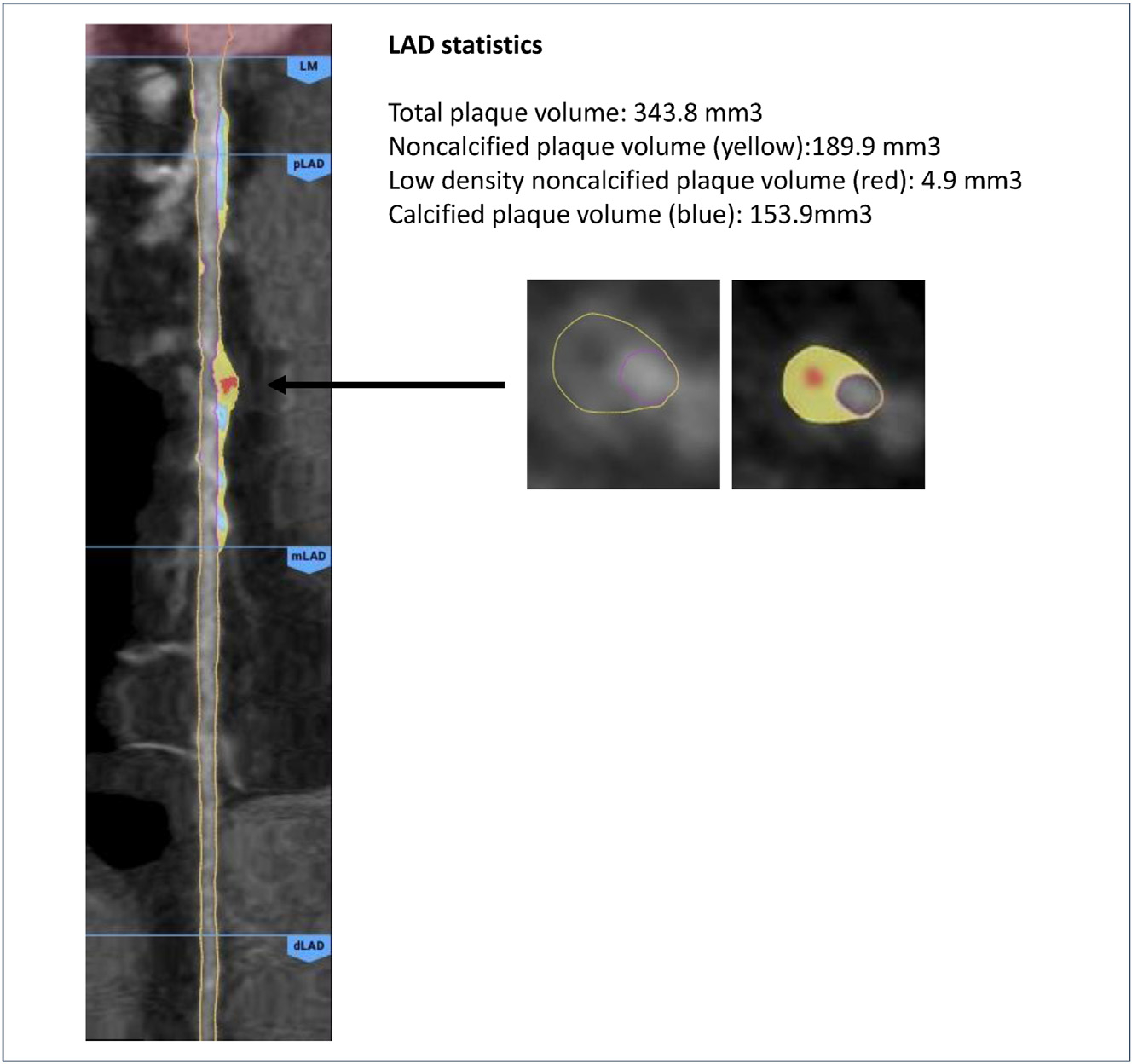
Multiplanar reconstruction of quantification of a left anterior descending coronary artery (LAD).

Imaging is performed with a CT scanner of ≥64 detector rows. Specific imaging parameters are expected to vary for each type of scanner and by site. Nitroglycerin and, under certain circumstances, beta blockers are recommended prior to CT acquisition in line with the standards of the Society of Cardiovascular Computed Tomography Guidelines for image acquisition.^24^ Scanning parameters, including gating, kVp, mA, and collimation are recommended to be optimized to achieve robust image data and maximum image quality whilst avoiding unnecessarily high radiation exposure as guided by the ALARA (“As Low As Reasonably Achievable”) principle, as set by the standards of the Society of Cardiovascular Computed Tomography Guidelines for image acquisition.^24^ Scan parameters should be included for each patient and include scanner model, mA, kVp, and whether a single or dual-energy mode is used. All de-identified CCTA images will be transferred to the Imaging Core Laboratory, located at Cleerly (Denver, CO, US). Automated software will evaluate CCTAs by a standardized approach in a blinded fashion CCTA analysis will be prescribed in a manual of operations. Stenosis variables that will be derived are amongst others luminal or area diameter stenosis, minimal luminal area, or cross sectional information at the slice of maximal stenosis. Coronary plaque variables are amongst others total plaque volume, the compositional subtypes (low density noncalcified plaque, noncalcified plaque, or calcified plaque), or high risk plaque features. Vascular morphology features include lumen volume, vessel volume, and indices about the remodeling of the coronary artery. The information will be derived from individual CT slice basis up to patient level data. An example of arterial quantification is provided in Fig. 2.

### 2.9. Data management

The Sponsor will perform data management activities including documentation of the systems and procedures to be used. All e-case report form (eCRF) data collection will be performed through a secure web portal and all authorized personnel with access to the electronic data capture system must use an electronic signature access method to enter, review, or correct data.

The data will be subjected to consistency and validation checks within the electronic data capture system. Completed eCRF images with the date-and-time stamped electronic audit trail indicating the user, the data entered, and any reason for change (if applicable) will be archived at the Investigator’s site and a backup copy archived with Cleerly Inc.

Primary data collection based on source-documented hospital and/or clinic chart reviews will be performed clearly and accurately by the clinical site personnel trained on the protocol and eCRF completion. eCRF data will be collected for all patients that are registered into the study.

The sponsor will archive and retain all documents pertaining to the study for the time of the study under evaluation, and for lifetime during the post-study phase. The Investigator must obtain permission from the sponsor in writing before destroying or transferring control of any study investigation records.

### 2.10. Statistical methods

The primary analysis of CONFIRM2 will evaluate the prognostic ability of coronary stenosis, atherosclerosis, and vascular morphology information by quantitative assessment in a cohort of symptomatic or asymptomatic patients undergoing CCTA for evaluation of suspected CAD. Cox proportional hazard models will be used to make inference and describe the magnitude of the prognostic value of each predictor using hazard ratios. Kaplan-Meier curves for the entire cohort and stratified by covariate level (e.g. quartiles of total plaque volume) will provide estimates of the risk of events over time and visualization of the magnitude of the effect of covariate on event probability. Additionally, machine learning techniques will be used to generate a multivariable predictive model. The dataset will be split into a training subset and a validation subset. The test subset will be used to build the predictive model, including evaluating the performance of various machine learning models such as random forest. The validation subset will be used to measure the predictive performance of the resulting model, measured as area under the receiver operating curve. Any covariates with more than 15% missing data will be excluded from analysis. Covariates with missing data that is less than or equal to 15% may be imputed using multiple imputation as a sensitivity analysis to evaluate the effect, if any on the cox model and machine learning results. Subjects with incomplete event data will be censored at the last time they were known to be event-free. The risk of type I errors was set at p < 0.05. In order to reduce the chance of type II errors a sample size of 30,0000 pts was chosen based on empirical data in order to permit adequate subgroup analyses while minimizing beta errors.

## 3. Discussion

The multicenter, international, CONFIRM2 trial will evaluate multidimensional associations between phenotypical manifestations of cardiovascular disease diagnosed by CCTA, serum biomarkers, clinical patient information, clinical presentation and therapy changes over time in relation to outcome.

A unique aspect of this trial is the use of automated software solutions for assessment of coronary plaque, stenosis, vascular morphology and cardiac structures. This allows quantification of coronary atherosclerotic burden, and its compositional subtypes, and can gauge subtle temporal burden changes in patients from serial CT scans. As such, CONFIRM2 will evaluate the value of quantification of coronary atherosclerosis to refine risk stratification and prediction of future events in comparison to current clinical strategies that are mainly aimed at detecting anatomically “obstructive” stenoses.

There has been great interest over the last several decades to identify the “vulnerable plaque”, a precursor for future ACS, which may allow to apply preemptive interventional strategies.^25–28^ Histopathological studies have shown that most myocardial infarctions or sudden coronary death results from thrombotic vessel occlusion with underlying plaque rupture or plaque erosions.^29^ The plaques associated with these culprit lesions have a thin-fibrous cap with an underlying large inflamed necrotic core and have therefore been proposed as high risk plaques. This approach, however, has been unsuccessful to date and instead the concept of assessing the “vulnerable patient” has been advocated.^30,31^ In part, this may be due to the dynamism of atherosclerotic plaques and the morphologic changes over time that contribute to heightening or lowering the likelihood of any given plaque to destabilize and become culprit lesion.^31,32^

Despite the observations that most vulnerable plaques will not result in clinically apparent coronary syndromes, quantification of high-risk plaque provides incremental information in relation to the overall atherosclerotic burden. The ICONIC study (Incident COroNary syndromes Identified by Computed Tomography), a nested case-control study investigating the prognostic value of these plaques accounting for the denominator of total atherosclerotic burden using a matched population, observed high-risk plaque features such as low-attenuation plaque, positive remodeling, spotty calcifications to independently predict ACS beyond clinical risk factors and total atherosclerotic burden.^18^ Furthermore, in the SCOT-HEART study (Scottish Computed Tomography of the HEART) the burden of low-attenuation plaque was the strongest predictor of MI and cardiac death, beyond calcium score and maximal stenosis in the coronary tree.^19^ These findings stress the need for whole heart atherosclerosis imaging focused on plaque morphology and burden, rather than on stenosis evaluation alone. Identification of high-risk patients, who have not yet experienced a coronary event, helps to allocate resources to potentially prevent the significant mortality and morbidity associated with coronary artery disease. A focus on plaque burden alone, or calcified plaque burden as done with calcium score imaging, provides good prognostic value but is insufficient. Plaque burden and especially the calcified component of plaque increase strongly with age, and do not adequately respond to disease modifying interventions such as diet change, life style modification and statin use.^33–36^

Current lipid lowering therapies halt plaque progression and provoke morphological changes towards lower risk namely an increase in calcified plaque phenotypes (and a regression of non-calcified plaque).^37,38^ Also, regular-moderate to high-intensity exercise of >150 min/week or healthy diets have been associated with plaque changes.^39^ CONFIRM2 will provide detailed quantification of plaque on a patient, lesion, and voxel-level detail from the entire coronary tree using an AI-derived quantitative methodology. We aim to further understand the levels of risk for specific phenotypical manifestations of coronary artery disease, by focusing on plaque type and vessel morphology. We will further explore dynamic associations between medication usage and changes over time, serum biomarkers, and quantified atherosclerosis. This will allow us to identify patients at high-risk who benefit more from imposing early and more specific preventive interventions (e.g. lower LDL-c targets).^39^ Beyond assessment of atherosclerosis, CCTA also enables non-invasive whole heart quantification of extra-coronary manifestations of cardiovascular disease such as left ventricular dilatation, ventricular hypertrophy and scar tissue. Also, associations of structural disease assessment with non-coronary related outcomes such as stroke or heart failure will be explored. This comprehensive analysis of patient specific parameters of vulnerability will assist in advancing precise and personalized medicine.

## 4. Conclusion

CONFIRM2 is a unique study and one of the largest CCTA studies ever performed that will allow evaluation of multidimensional associations between phenotypical manifestations of cardiovascular disease by CCTA using machine-learning solutions, and static and time varying changes in clinical information, cardiac imaging and clinical outcomes. This will further refine risk stratification providing an impetus to precision medicine in CAD patients.

## Figures and Tables

**Table 1:** Inclusion and exclusion criteria.

Inclusion criteria	Exclusion criteria

Age ≥18 years	Individuals unable to provide informed consent (prospective cohort)
Undergoing clinically indicated CCTA with ≥64- detector row CT	Absence of information for baseline clinical data and follow-up of clinical events
	Pregnancy Non-cardiac illness with life expectancy <2 years
